# Landscape Phage: Evolution from Phage Display to Nanobiotechnology

**DOI:** 10.3390/v10060311

**Published:** 2018-06-07

**Authors:** Valery A. Petrenko

**Affiliations:** Department of Pathobiology, Auburn University, Auburn, AL 36849-5519, USA; petreva@auburn.edu; Tel.: +1-334-844-2897

**Keywords:** phage display, landscape phage, major coat protein, nanomedicine, diagnostics, biosensors

## Abstract

The development of phage engineering technology has led to the construction of a novel type of phage display library—a collection of nanofiber materials with diverse molecular landscapes accommodated on the surface of phage particles. These new nanomaterials, called the “landscape phage”, serve as a huge resource of diagnostic/detection probes and versatile construction materials for the preparation of phage-functionalized biosensors and phage-targeted nanomedicines. Landscape-phage-derived probes interact with biological threat agents and generate detectable signals as a part of robust and inexpensive molecular recognition interfaces introduced in mobile detection devices. The use of landscape-phage-based interfaces may greatly improve the sensitivity, selectivity, robustness, and longevity of these devices. In another area of bioengineering, landscape-phage technology has facilitated the development and testing of targeted nanomedicines. The development of high-throughput phage selection methods resulted in the discovery of a variety of cancer cell-associated phages and phage proteins demonstrating natural proficiency to self-assemble into various drug- and gene-targeting nanovehicles. The application of this new “phage-programmed-nanomedicines” concept led to the development of a number of cancer cell-targeting nanomedicine platforms, which demonstrated anticancer efficacy in both in vitro and in vivo experiments. This review was prepared to attract the attention of chemical scientists and bioengineers seeking to develop functionalized nanomaterials and use them in different areas of bioscience, medicine, and engineering.

## 1. Introduction

Filamentous bacteriophages (shortly phages), remarkably tolerant of dramatic structural alteration, are in every respect the perfect material for bioengineering. The structure of phage capsids is encoded in small single-stranded circular DNA and can be reengineered using common DNA techniques. Their capsids have a well-defined architecture, consistently adaptable for nanofabrication. The precise structures of filamentous phages and their coat proteins have been determined, allowing for the engineering of variants with precise arrangements of fused functional peptides and desired shapes and functions of phage-originated nanocomposites. The development of phage-functionalized biosensors and phage-targeted nanomedicines, which is the focus of this review, has been closely linked to the advances in structural phage biology and phage display technology. For a complete summary of Ff phage structure and biology, one can be referred to comprehensive reviews [1,2]. This review focuses on Ff phage display vectors, as precursors of landscape phages and as major workhorses in phage nanobiotechnology. It considers also those aspects of the structure and life cycle of Ff phages that are of direct practical interest to chemical scientists and bioengineers seeking to develop phage virions as specifically functionalized nanoparticles and use them in different areas of bioscience, medicine, material science, and engineering, as summarized in [3].

## 2. The History of Filamentous Phages

**Figure 1:**
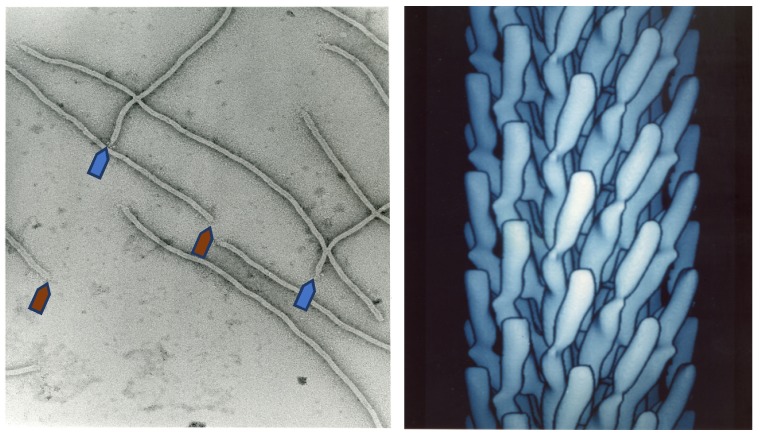
Electron microscopy image of filamentous phage (**left**) and electron density model (**right**) of filamentous phage M13 (Courtesy of Lee Makowski and Gregory Kishchenko. Adapted from [8]). Blue and red arrows depict the sharp and blunt ends of the phage capsid with attached minor coat proteins pIII/pIV and pVII/pIX, respectively (five copies each). Major coat protein (~2700 copies) forms the tubular capsid around viral single-stranded DNA.

The story of bacteriophage discovery is told by Stent in his book *Molecular Biology of Bacterial Viruses* [4]. After French microbiologist Félix d’Hérelle introduced phages as antibacterial therapeutic agents, phage therapy soon became a boom and brought about great optimism in biomedical scientists [5]. After the discovery of penicillin in 1928 [6] and its triumphal introduction in the 1940s, the interest in the practical applications of phages as antibacterial agents faded, along with increasing interest in phages as tools for fundamental biological studies [7]. On this new wave of “phage renaissance”, in the early 1960s, a group of filamentous phages (M13, fd, and fl) infecting conjugative F-pilus-positive *Escherichia coli* strains were isolated from sewage systems in the United States and Europe [1]. These small male-specific filamentous coliphages incorporate a single-stranded circular DNA encapsulated in a rigid tubular capsid (Figure 1). Because genomes of these phages code for a small number of proteins, reproduction of the phages is mostly supported by host bacteria. For this and other reasons, the small DNA phages became very popular model systems in molecular biology and versatile tools in genetic engineering and biotechnology.

## 3. Phage Engineering

In the mid-1970s, as a result of the development of the gene-splicing technique, the creation of molecular chimeras became a novel stirring area in molecular biology and biotechnology [9]. For a comprehensive presentation of the most commonly used molecular cloning systems, the reader is referred to [10]. As oligonucleotide-directed mutagenesis and DNA sequencing grew into routine genetic engineering techniques in the 1970s [9], filamentous phages emerged as unique DNA cloning vectors, very convenient for gene reconstruction and protein engineering (reviewed in [11]). These vectors provide a researcher with one of the two strands of vector DNA in a very useful form—easily purified phage particles. The extracted single-stranded DNA serves then as a template for sequencing and site-directed mutagenesis. The extendable tubular capsid of filamentous phages, oppositely to spherical capsids, readily accommodate enclosed recombinant DNA of any length [2].

In 1985, the emerged filamentous-phage recombinant DNA technique was adapted to design a novel type of viral chimera that inspired the development of phage display technology [12]. To construct this type of viral chimera, an alien DNA fragment is inserted into a gene encoding one of the phage coat proteins in such a way that the encoded “foreign” peptide is genetically fused to a coat protein and, by these means, exposed on the surface of the phage particle. A phage display library is a collection of fusion phage particles, containing diverse foreign DNA sequences and displaying diverse fusion peptides on the surface. The inserted foreign DNA can be derived from a natural source or it can be chemically synthesized. This technique has allowed for the constructing of phage libraries displaying billions of random peptides on the phage surface, as reviewed in [13]. Surface exposure of guest peptides presents a possibility of affinity selection, an essential aspect of phage display technology [12]. Affinity selection of phages exemplifies a kind of natural selection or molecular evolution in vitro, in which the phage library is comparable to a population of biological species and affinity for the receptor is equivalent to the “fitness” that aids an individual organism to survive and produce more offspring in the next generation. General principles and numerous applications of phage display technology are covered in several books and reviews, for example [12,13,14,15,16]. Here, the focus is on the logic and advances of the landscape phages—multivalent p8-type filamentous-phage constructs, which stand out from traditional phage display systems as a result of their unique structure and emerging properties [17]. Because of these characteristics, landscape phages can be of major interest as a foundation of phage nanobiotechnology—a new area of material science and nanotechnology. Many other filamentous phage vectors, suitable for other types of phage display systems (see below), are presented in [15,18].

## 4. Classification of Filamentous Phage Display Systems

**Figure 2:**
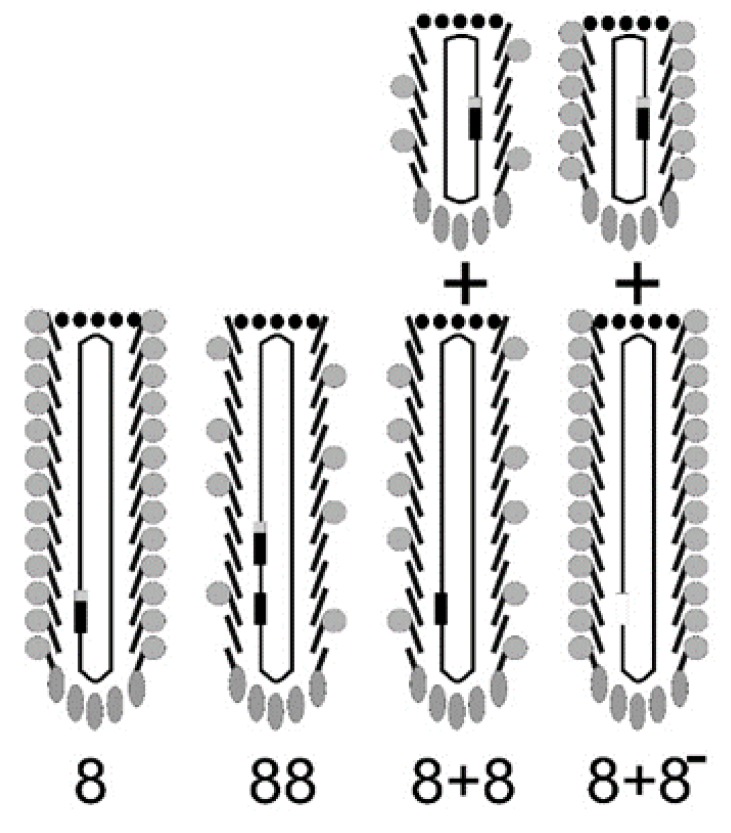
A type 8 vector (landscape-phage vector) contains multiple cloning sites in a single gene *gpVIII*. A type 88 vector harbors two genes: *VIII*, the wild-type *gpVIII*, and a recombinant rec-*gpVII*I that has the cloning sites. Type 88 phage capsids are composed of a mosaic of wild-type and recombinant pVIII proteins. In type 8 + 8 systems, the recombinant version and the wild-type version of gene *VIII* are on separate genomes: on a phagemid and on a helper phage ([11,27]). As for type 88 phage particles, the helper and phagemid virions in the type 8 + 8 system have a mosaic composition of recombinant and wild-type pVIII. In the type 8 + 8^−^ system, the helper phage lacks *gpVIII*, and all pVIII proteins are recombinant, as in the type 8 vector.

**Figure 3:**
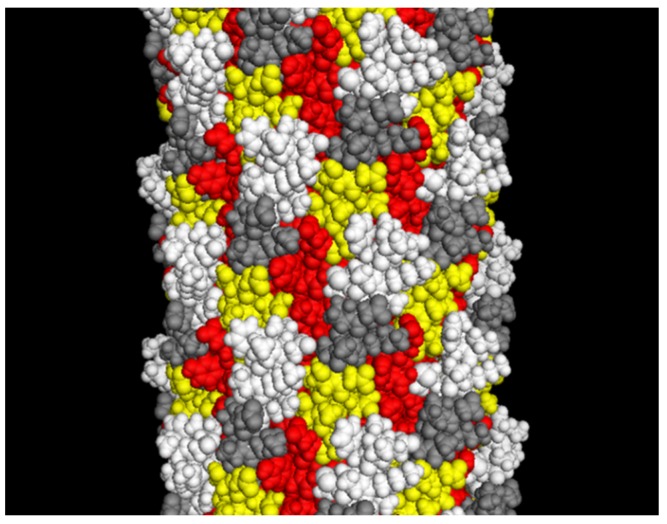
The landscape phage model (~10 nm segment of the full length). White: depicted atoms belong to the foreign peptides; yellow: pictured random amino acids in the phage α-library [28]; red: depicted amino acids belong to a small displayed segment of the hydrophobic domain predominantly buried in the capsid; grey: depicted amino acids belong to a “conservative” small segment of amphipathic domain. Adapted from [20].

To compare the landscape phage with other types of filamentous phage display systems, it is worth introducing a classification of phage display constructs [12,18]. Some display systems, mainly pIII-type, are mostly monovalent, with one or even fewer peptides displayed per phage particle. The monovalent type of display is advantageous when the goal is a high monovalent affinity of displayed peptides to the target. Multivalent phage display vectors belong to the group *n* that includes type 3, 8, 6, 7, and 9 systems, for which the foreign peptide is fused to all copies of the pIII, pVIII, pVI, pVII or pIX protein. The phage vector genome harbors a single recombinant coat protein gene (*III*, *VIII*, *VI*, *VII*, or *IX*)*.* Landscape phages, which are constructed using type 8 vectors (Figure 2), are an extreme variant of multivalent display, in which thousands of fusion peptides are arranged in an ultra-high-density array on the surface of the virion (Figure 3). The dramatic alteration of the phage architecture can lead to emergent properties of the whole particle that are not directly linked to the properties of the individual fusion phage proteins and inserted peptides, as was observed in [19]. Multivalent display in p8-type phage vectors (named the landscape phage) [17] undermines selection for high affinity by disregarding individually weak and strong peptide ligands, which cannot be distinguished between because of greatly increased avidity (effective affinity). There are many applications, however, in which multivalent display is a distinctive advantage. As exemplified below, polyvalent display in landscape phages is particularly effective in the engineering of dense, ideally organized, robust interfaces in biosensors [20,21]; as diagnostic and therapeutic probes for targeting multiple cellular receptors [22,23,24,25]; as leads for vaccine development; and for the assembly of a broad spectrum of new materials [26].

The first *n*-type pVIII phage display vectors [29] originated from the recombinant phage fd-tet [30]. They were the basis of the first peptide and antibody libraries [31,32,33,34] and were employed in many different projects (for a review, see [12]). The remarkable feature of the fd-tet vectors is a small number of double-stranded phage replicative form (RF) DNA molecules per cell, because of a defect in the origin of replication that forces RNA polymerase to initiate the synthesis of a DNA replicative form using inefficient alternative initiation sites. Because of its replication defectiveness, the fd-tet vector produces in infected bacteria just several copies of RF DNA, which is hardly sufficient to support the phage assembly but allows the prevention of “cell killing” caused by toxic effects of excess fusion proteins.

**Figure 4:**
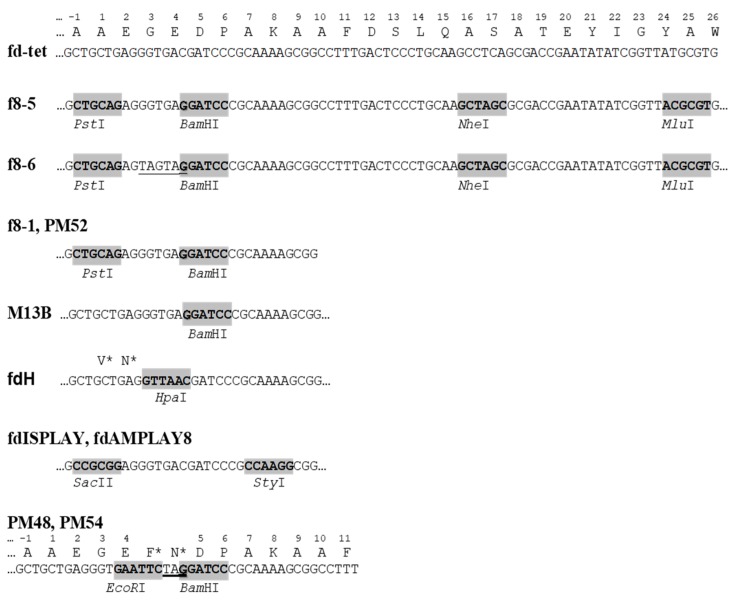
Cloning sites in p8-type filamentous phage vectors. Stop codons are underlined; asterisks mark amino acids synthesized in repressor strains.

Table 1 exemplifies the type 8 phage display vectors. The most advanced vectors f8-5 and f8-6 are suitable for the insertion of random peptides into the pVIII protein using *Pst*I, *Bam*HI, *Nhe*I, and *Mlu*I sites [27,35] (Figure 4). The presence of TAG amber stop codons between the cloning sites *Pst*I and *Bam*HI in the f8-6 vector precludes the synthesis of the wild-type vector in a non-suppressor strain, avoiding contamination of the library with vector phages.

## 5. Development of Landscape-Phage Libraries

**Figure 5:**
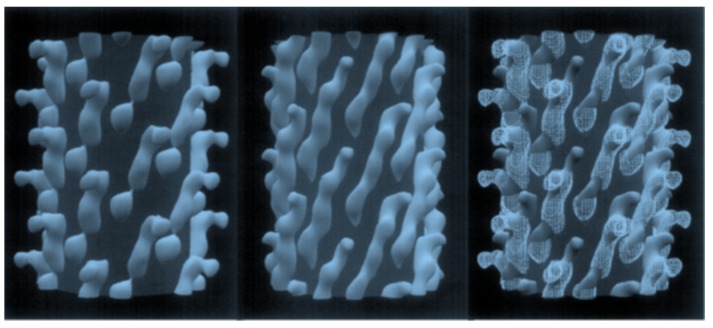
Configuration of a 5-mer peptide displayed on bacteriophage (phage) M13. Computer rendering of a ~10 nm length of the surface of electron density maps of M13 (**left**), fusion phage with 5-mer peptide inserted in all copies of p8 proteins (**center**), and a rendering of the differences between images (**right**). A cylinder of 2.5 nm radius was added to images to mask essentially identical interior features of the phages. About half of each coat protein is visible in phage surface images. Adapted from [48].

“To the surprise of most phage biologists” [2], short guest peptides were displayed on the N-terminus of the major coat protein pVIII, shortly after the phage display on the coat protein pIII was developed [42,43,44]. The design of the first type 8 phage display constructs was motivated mostly by the interest of researchers in universal polyvalent vaccines and artificial immunodiagnostics; the foreign peptide was thus an epitope mimic interacting with an antibody [38,43,45,46]. They were constructed using the p8-type phage vector M13B [47] (Table 1; Figure 4). Phage particles displayed the foreign 5-mer peptide on every pVIII subunit [38,43], which led to the increasing of the phage mass by 10% (Figure 5).

**Figure 6:**
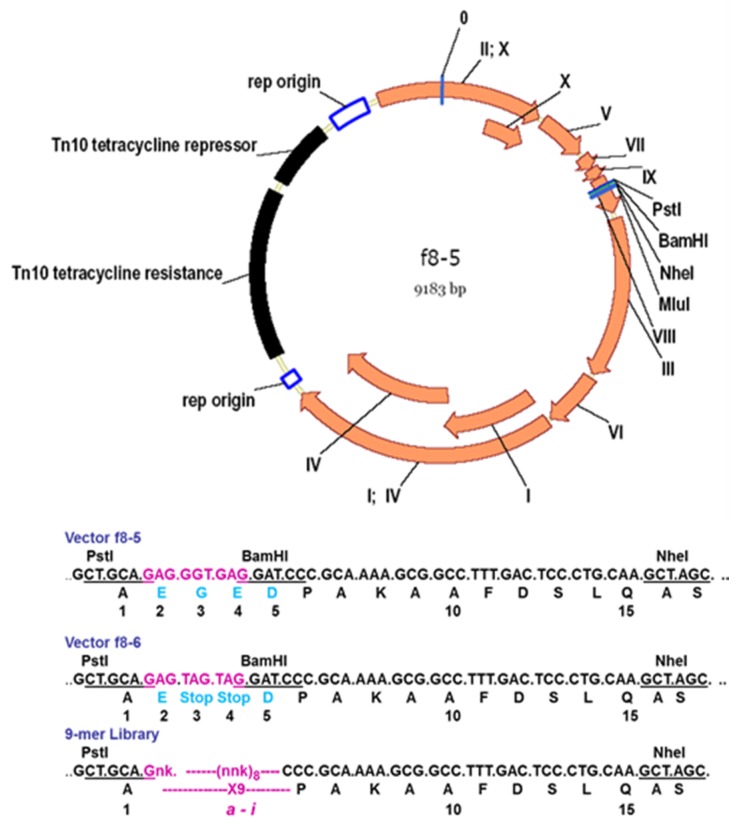
Phage display peptide libraries for vectors f8-5/f8-6 and f8-6. Characteristics of f8-6 vector: Restriction sites *Pst*I, *Bam*HI, *Nhe*I, and *Mlu*I in *pVIII* gene; Tet resistance gene spliced in origin of replication; low copy number of RF DNA and phages/cell; formation of tiny plaques on Tet-negative and colonies on Tet-positive agar plates.

**Figure 7:**
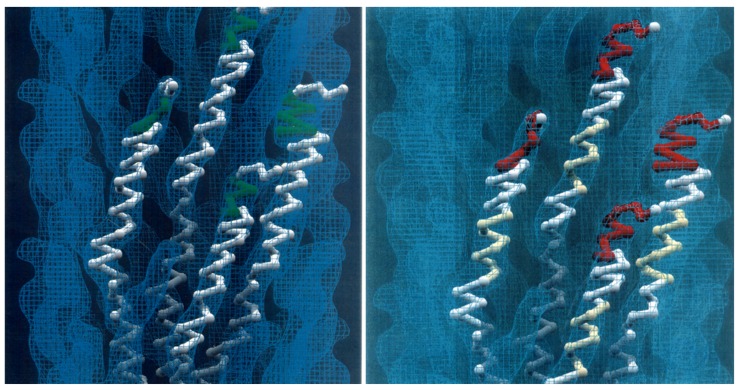
Models of landscape-phage libraries. **Left**: Ball-and-stick model of four neighboring pVIII proteins with inserted 5-mer peptides (green). About 1% of the phage length is shown. **Right**: Ball-and-stick model of three neighboring pVIII proteins with N-terminal random foreign 8-mer peptides (red) and mutagenized central segments (yellow) on the surface of the phage capsid (blue contour). The courtesy of Lee Makowski and Gregory Kishchenko.

Surprisingly, such phage particles, with a dramatic distortion of their surface landscape, can infect *E. coli* and replicate in bacteria to produce plentiful progeny phage particles. Such phage particles were named the landscape phage to highlight the uniqueness of their architecture formed by thousands of the guest peptides displayed in a compact, reiterating pattern over the whole length of the phage capsid [17,25,27,41,49], as illustrated in Figure 3 and Figure 5. The structures of these chimeric phages and fusion viral p8 proteins were studied, in comparison with the wild-type phage fd, by low- and high-resolution physical methods [44,48,50]. It was found that the inserted 5-mer peptides are fused to each p8 subunit and agglomerate on the surface of the phage. It was observed, however, that the size and structure of alien peptides, fused to the N-terminus of each phage protein p8, are restricted [36]. For example, the filamentous phage f1 readily tolerates a high diversity of 6-mer peptide fusion (resulting in an increase in the length of the major coat protein pVIII from 50 to 57 amino acids), but only a portion of phages are infective with longer fusions. As was exemplified, 40% of random 8-mer (59 amino acid long pVIII), 20% of 10-mer (61 amino acid long pVIII), and 1% of 16-mer (67 amino acid long pVIII) survived. The replacement of the M13 vector for the low-copy-number vector f8-1 (Table 1) allowed for the construction of the first multibillion-clone 8-mer landscape-phage display library f8/8 [11,17] (Figure 4; Table 1). To overcome limitations in the sizes of foreign peptides, we used a novel *replacement* strategy that combines the insertion of new peptides with the deletion of peptides that are not essential for phage viability. Thus, in constructing the first landscape library (named the f8/8 library) using the f8-1 vector, amino acids 2–4 (EGE) at the N-terminus of the mature pVIII protein were deleted and replaced by random 8-mers. As a result, the length of the phage fusion pVIII protein expanded only by five amino acids. In the f8/9 library, prepared using vector f8-6 (Figure 6), the amino acids EGED were substituted by random 9-mers. During this reconstruction of the pVIII protein**,** its length expanded to 55 amino acids, as in phage particles displaying foreign 5-mers [38,43] and in the f8/8 library [17] (Figure 7). As could be expected, the replacement of the negatively charged peptide EGDD for random peptides led to dramatic changes in the phage’s physico-chemical and biological properties and, accordingly, to the censoring of the phage libraries’ diversity, as demonstrated in [35].

Taking into account that the filamentous phage fd-tet can tolerate extension of the length of the major coat protein to 59–61 amino acids [36], one can propose that landscape-phage libraries with random 12–15-mer guest peptides can be designed using our replacement strategy. In particular, the performance of this strategy was confirmed by the construction of a conformationally constrained half-billion landscape-phage library, which displayed mutagenized amino acids 12, 13, 15–17, and 19 of the α-helical amphipathic domain of the pVIII protein [28], as portrayed in Figure 7. Such a combination of constrained and flexible random peptides grafted into the phage scaffold can mimic the architecture of antigen binding sites—regions in antibodies containing a number of highly mutable peptide loops, about 10 amino acids in length each, called *hypervariable regions* or *complementarity-determining regions* (CDRs) [51]. Indeed, in experiments with representative antigens, it was shown that landscape phages can serve as a new kind of substitute antibody that binds target antigens with a high affinity, specificity, and selectivity [49]. As substitute antibodies, landscape phages recommended themselves very well in biosensors and other analytical devices, as described below.

The multivalent display of peptides in p8-type landscape phages provides abundant freedom in engineering robust phage nanomaterials with rationally designed or selectable properties, as well as platforms for biospecific interactions. The phage-decorating foreign peptides constitute up to 25% of a phage particle’s weight and cover about 50% of its surface (Figure 3). Because of the strong avidity revealed by the interaction of landscape phages with various biomolecules and nanomaterials, they are commonly used as substitute antibodies with subnanomolar effective affinities and high specificity for counterpart antigens. As antibody mimics, landscape phages overcome some intrinsic restrictions of antibody technology. Landscape phages are easily obtained from discontinuously growing bacteria—an effective recombinant protein manufacture system. The robust landscape phage shows an indefinite shelf life, without losing infectivity or specific functionality. These and other characteristics attest that landscape-phage libraries are an ideal rich source for specifically functionalized filamentous nanomaterials and recombinant peptide-fusion phage proteins. Landscape-phage probes have been discovered for a range of organic and inorganic materials that comprise widespread complexity [8,20]. They efficiently serve as substitutes for antibodies against bacterial and cancer cells in biosensors and targeted drug/gene delivery vehicles [49,52,53,54,55,56], as is exemplified below.

## 6. Landscape-Phage-Based Biosensors for Detection Monitoring of Biological Threats

Medical manifestations of disease, confirmed by biochemical, microbiological, and animal tests, remain the gold standard in clinical diagnostic laboratories. Currently, new requirements for fast, sensitive, accurate, and inexpensive detection platforms devalue the traditional detection methods. Modern immunoassays and biosensors require a biorecognition probe, which is attached to the interface of the analytical device, binding the target biological or chemical threat agent and generating a measurable signal. Most analytic platforms rely on the use of monoclonal antibodies as biorecognition probes. However, their broad application is limited by high cost, low specificity, less-than-optimal affinity, and sensitivity to environmental stresses. Landscape-phage display is a novel concept that allows for the development of diagnostic and detection interfaces that meet modern criteria for biological detection and monitoring [20,21,49,52,57]. It was proved that the landscape libraries represent an inexhaustible rich source of substitute antibodies—filaments that bind protein and glycoprotein antigens with nanomolar affinities and high specificity [49]. Landscape-phage-based interfaces performed in many ways much more effectively than their natural immunoglobulin counterparts in the same detector platforms. Target-specific landscape-phage probes can be prepared as described in commonly available protocols [58,59]. In my laboratory, *Bacillus anthracis* spore-specific landscape phages were selected by incubation of the landscape-phage library with spores immobilized in wells of enzyme-linked immunosorbent assay (ELISA) plates. Non-related phage particles were discarded, and spore-bound particles were released with acid buffer and collected [60]. To discover phage probes against the bacteria *Salmonella typhimurium*, a similar panning procedure was used, along with a coprecipitation procedure, in which complexes of bacterial cells and phage particles were isolated by centrifugation [61]. After titering of the phage in the host *E. coli* strain, random phage clones were used for the identification of selected displayed peptides by sequencing of recombinant *gpVIII* DNA [61]. The specificity of the phage probes selected against the *B. anthracis* spores was confirmed by ELISA, in which a phage-coated microtiter plate was incubated with spores and then treated with *B. anthracis* specific antibody. Similarly, the specificity and selectivity of phage probes against *S. typimurium* were confirmed by phage ELISA [61]. The established selection methods were successfully used for the discovery of diagnostic probes against classical swine fever virus [62], *Vibrio parahaemolyticus* [63], and *Staphylococcus aureus* [64]. For comprehensive reviews on phage-based pathogen biosensors, one can be referred to [21,58]. Data on the performance of landscape-based interfaces in different biosensor platforms are summarized in Table 2.

From these summarized data, one can conclude that landscape-phage display technology allows for the construction of libraries of diverse nanostructures boarding on the phage capsid—an enormous reserve of probes for the preparation of diagnostic and detection interfaces in analytical and biosensor platforms. Landscape phages may also serve as unique robust and inexpensive molecular recognition interfaces for field-use detectors and real-time monitoring devices for the control of biological and chemical threats. The commercialization of landscape-phage-based analytical interfaces may significantly enhance the performance of commercially valuable biosensors.

## 7. Diagnostic-Therapeutic Cancer Cell-Targeted Landscape

Targeted nanomedicines suggest a less toxic variant for cancer patients by reducing the drug supply to non-cancer tissues and enhancing their accumulation in tumors. The development and screening of targeted nanomedicines were recently facilitated by introducing the methods of high-throughput selection of cancer cell-binding landscape-phage proteins and their self-assembly into drug-loaded nanovehicles. These applications of the landscape-phage technology resulted in the development of diagnostic probes and targeted nanomedicines towards human prostate, breast, lung, pancreatic, and colorectal carcinoma cells [69,70,81,82,83]. The major paradigm that preceded the development of the landscape-phage technology was that phage-derived analytical and medical devices have to recognize the same antigens and receptors, which serve as targets in the course of phage selection [53,55,84,85]. Indeed, the cell-interacting specificity of landscape-phage particles and their isolated proteins was adequately translated to the phage protein-targeted nanomedicines, improving their cytotoxic efficacy towards cancer cells both in vitro and in vivo [8,59,86,87,88,89,90,91,92,93,94,95,96,97,98]. 

**Figure 8:**
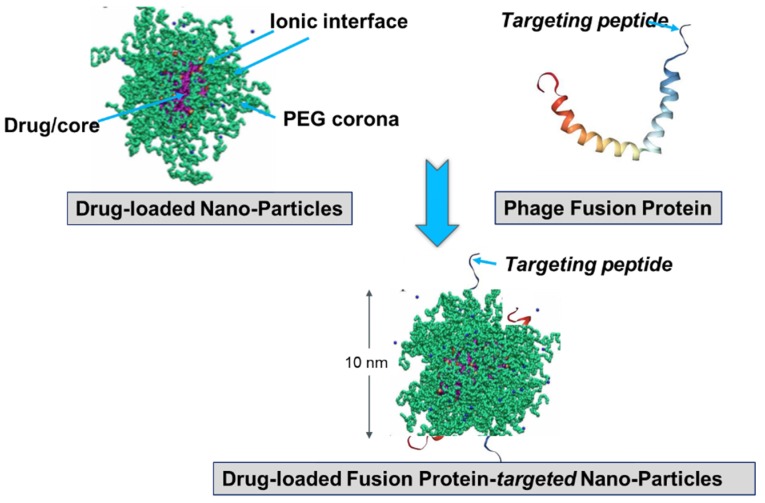
Preparation of landscape-phage pVIII-targeted paclitaxel-loaded PEGylated lipid micelle particles [94,98]. Equilibrated micelles contain three distinctive regions: lipid hydrophobic core, ionic interface, and poly(ethylene glycol) (PEG) corona. The fusion pVIII spans the core and displays the foreign peptide in PEG corona. The insoluble in water drug (paclitaxel) is solubilized in the core. Image of PEG-ylated sterically stabilized micelles is adopted from [104]. The model of pVIII phage protein in lipid environment is adapted from [105].

It was presumed that not only the tumor-targeting specificity of landscape phages but also their specific immunogenicity could be translated to the phage-driven nanomedicines. While intrinsic immunogenicity of landscape phages can be favorable, for example, in the construction of phage-based vaccines [12,14,43,99], it can create serious concerns about the efficacy and safety of phage-derived nanomedicines, which is equally true for many biologics [100]. To reduce the potential immunogenicity of phage-targeting micelles and liposomes, PEGylation was used to prevent nanoparticle aggregation and thereby mask antigenic epitopes, as illustrated in Figure 8. Interestingly, the phage protein-fusion peptides sheltered in the poly(ethylene glycol) (PEG) corona demonstrated expected targeting effects and profound anticancer cell activity both in vitro and in vivo [82,83,85,87,89,92,93,94,95,96,98].

**Figure 9:**
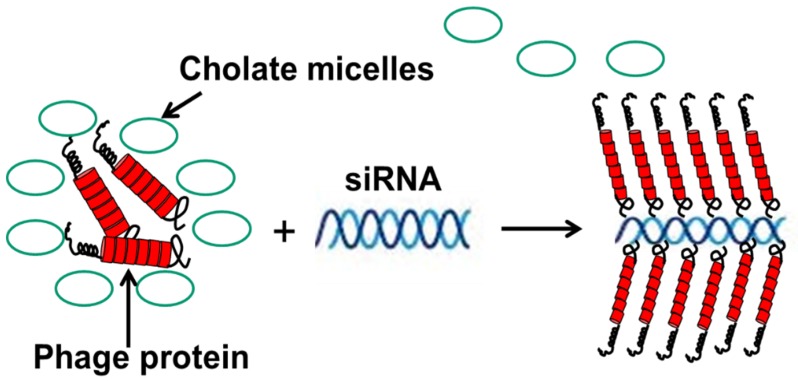
Preparation of nanophages from fusion phage protein and siRNA. The complex is formed during removal of protein-stabilizing cholate buffer. After the removal of cholate, the complex of RNA and protein is stabilized as a result of (a) the hydrophobic interaction of protein subunits, and (b) electrostatic interaction of positively charged C-terminus of the major coat protein and negatively charged phosphates of siRNA. Displayed N-terminus of phage protein serves as targeting ligand, which brings siRNA into the breast cancer cells. Adapted from [86].

The fusion major coat protein pVIII is the universal construction material that can be used in the preparation of cancer cell-targeted nanomedicines and diagnostics. The protein contributes ~90% to the virion mass [55] and can be easily isolated from phage particles in pure form using fast and simple procedures [55,85]. As a natural multifunctional membrane protein, pVIII readily associates with lipid nanoparticles, such as micelles and liposomes [54,90,101]. Its spontaneous insertion into lipid membranes (Figure 8), which is controlled by hydrophobic and electrostatic interactions and driven by electrophoretic forces, is discussed in [55]. The major coat protein was used for the targeting of liposomes [81,82,87,89,90,92,93,95,96] and micelles [81,83,94,98], as well as for the encapsulation of DNA and RNA [86,102] (Figure 9). The later method mimics the natural ability of the phage coat protein to encapsulate its genomic DNA during phage reproduction [101,103]. As noted above, the proficiency of the selected landscape phage to bind and penetrate into cancer cells is translated both to their individual proteins [91] and to phage protein-targeted nanomedicines. This was proved, for example, in our experiments with paclitaxel-containing micelles targeted with phage fusion protein specific for human breast cancer cells. It was shown that nucleolin-targeted micelles bind to their cognate target cells and demonstrate a significantly higher specific cytotoxicity towards target cells in comparison with non-targeted micelle nanomedicines. Thus, cancer cell-specific phage proteins obtained by phage selection from landscape phage libraries demonstrated a high potential as a substitute antibody for polymeric micelle-based pharmaceutical preparations [92,93,94,95,97,98].

In another scenario, fusion phage proteins were used as nanocarriers for the targeted delivery of siRNA to breast cancer cells [86]. This approach mimics the assembly of phage protein and phage DNA during different steps of the phage life cycle—and more specifically, the encapsulation of the nucleic acid into the phage capsid during secretion of the phage from the cell membrane of the host bacterium. Similarly, phage fusion proteins form a tubular vehicle for the accommodation and targeting of nucleic acids, such as siRNA, to form nanoparticles (11 nm diameter), called nanophages (Figure 9). In nanophages, the breast-cancer-targeting fusion phage protein not only protects siRNA from nucleases, but at the same time directs the delivery of a siRNA cargo to the target breast cancer cells and mediates knockdown of the targeted gene. Thus, nanophages exemplify elegant multifunctional vehicles for the targeted delivery of therapeutic nucleic acids to the site of disease.

## 8. Conclusions

In contrast to the most commonly used p3-type phage display vectors, which were designed to discover a desired phage-displayed peptide, the p8-type landscape-phage technology was developed with the goal to modify the properties of the whole fusion phage particle. In landscape-phage libraries, introduced in this review, a significant portion of the virion’s mass (~10%) and surface (~25%) varied between distinct phage clones, comprising a billion-clone population of phage particles with unexpected emergent properties. The landscape-phage-based methodology for engineering of novel materials and devices exploits very specific mechanisms of phage biosynthesis, involving the precise self-assembly of phage particles. Landscape-phage libraries are a rich reservoir of already functionalized landscape phages and their proteins, which can be readily discovered using advanced methods of selection. Using landscape phages and phage fusion proteins for the construction of nanodevices allows for bypassing the critical and troublesome conjugation steps. No re-engineering of the landscape phage is required, as the phage particles are ready to be used as they are or can be transformed into various phage-programmed biospecific materials. The concept of a landscape phage has already contributed to various areas of biomedicine and nanotechnology, including the development of novel biosensors and monitoring devices, imaging systems, targeted nanomedicines, gene delivery platforms, universal vaccine leads, materials for bone and tissue repair, and so forth. I hope that this review will help chemists, biochemists, and bioengineers stay up-to-date with current trends in phage display and phage nanotechnology and encourage them to find new avenues in realization of their goals.

## Figures and Tables

**Table 1:** Type 8 vectors.

Name	Parent Phage	Antibiotic Resistance	Applications	Reference
f8-1	fd-tet	Tet	Billion-clone 8-mer peptide library	[17]
f8-5	fd-tet	Tet	Hundred-million-clone α-helical peptide library	[27]
f8-6	fd-tet	Tet	Billion-clone 9-mer peptide library	[35]
PM54	fd-tet	Tet	Small 6–16-mer peptide libraries	[36]
PM52	fd-tet	Tet	Small 6–16-mer peptide libraries	[36]
fdAMPLAY8	fd	Amp	Cloning of peptides	[37]
fdH	fd	None	Cloning of 4- and 6-mer peptides	[38]
fdISPLAY	fd	None	Cloning of peptides	[39,40]
PM48	f1	None	Ten-million-clone 8-mer peptide library; small 9-mer library	[36,41]
M13B	M13mp10	Amp	Cloning of 5-mer peptides	[42,43]

Tet: Tetracycline; Amp: ampicillin.

**Table 2:** Performance of landscape-phage-based interfaces.

Biosensor	Interface	Analyte	Sensitivity, Detection Range	Reference
Quartz crystal microbalance (QCM*)*	Phage coupled with phospholipid via biotin-streptavidin	β-galactosidase from *Escherichia coli (*β-gal)	Kd = 0.6 nM	[57]
	Phage immobilized by physical adsorption	β-gal	Kd = 1.7 nM	[65]
		*Salmonella typhimurium*	100 cells/mL	[66]
Surface plasmon resonance (SPR) spectroscopy	Phage immobilized by physical adsorption	β-gal	1 pM to 1 nM	[67]
	Enhanced green fluorescent protein (eGFP)	Phage in solution used in competition assay	1.2 × 10^−14^ M (in competiton assay)	[68]
Electrochemical impedance cytosensor	Phage immobilized on the electrode surface by physical adsorption	Colorectal carcinoma cells	79 cells/mL, 2 × 10^2^–2 × 10^8^ cells/mL	[69,70]
	Conjugate of the hybrid (8 + 8)-type M13 phage and electronically conductive polymer	Human serum albumin (HSA)	100 nM to 5 µM	[71]
Magnetoelastic particle resonators	Phage immobilized by physical adsorption	*Bacillus anthracis* spores	10^2^–10^3^ cfu/mL	[21,72,73]
		*S. typhimurium*	10^2^–10^4^ cfu/mL	[74]
Magnetoelastic microcantilever	Phage immobilized by physical adsorption	*S. typhimurium*	Not determined	[75]
Colorimetric immunoassay	Conjugate of pVIII fusion protein and cysteamine (CS)–gold nanoparticles (CS–AuNPs)	*Staphylococcus aureus* (*S. aureus*)	19 cfu/mL/mL	[64]
	Conjugate of pVIII fusion protein and protein–MnO_2_ nanosheets	*Vibrio parahaemolyticus*	15 cfu/mL, 20–10^4^ cfu/mL	[63]
Enzyme-linked immunosorbent assay (ELISA)	Phage immobilized by physical adsorption	*B. anthracis* spores	Not determined	[60]
		β-gal	Kd = 30 nM	[49,57]
		Neutravidin	Not determined	[49]
		Streptavidin		[49]
		Antibodies against gonadotropin-releasing hormone (GnRH) in patient sera		[76]
		Lyme disease patient sera		[77]
		Free prostate-specific antigen (f-PSA)	0.16 ng/mL, 0.825–165 ng/mL	[78]
		Total prostate-specific antigen (t-PSA)	1.6 ng/mL, 3–330 ng/mL	[79]
Differential pulse voltammetry (DPV) analytical platform	Phage conjugated to the gold electrochemical immunosensor		3 pg/mL, 0.01–100 ng/mL	
Phage microarray	Phage conjugated with NHS-functionalized slide	Cellulytic endoglucanase I (EG I)	5–500 nM	[80]
